# Adiponectin: An Attractive Marker for Metabolic Disorders in Chronic Obstructive Pulmonary Disease (COPD)

**DOI:** 10.3390/nu5104115

**Published:** 2013-10-14

**Authors:** Andrea Bianco, Gennaro Mazzarella, Viviana Turchiarelli, Ersilia Nigro, Graziamaria Corbi, Olga Scudiero, Matteo Sofia, Aurora Daniele

**Affiliations:** 1 Department of Medicine and Health Sciences, University of Molise, Via Giovanni Paolo II, Loc. Tappino, Campobasso 86100, Italy; E-Mails: viviana.turchiarelli@unimol.it (V.T.); graziamaria.corbi@unimol.it (G.C.); 2 Department of Cardiothoracic and Respiratory Sciences, Second University of Naples, Via Leonardo Bianchi, Monaldi Hospital, Naples 80131, Italy; E-Mail: gennaro.mazzarella@unina2.it; 3 CEINGE Biotecnologie Avanzate Scarl, Via Gaetano Salvatore 486, Naples 80145, Italy; E-Mails: nigro@ceinge.unina.it (E.N.); scudiero@ceinge.unina.it (O.S.); aurora.daniele@unina2.it (A.D.); 4 IRCCS–Fondazione SDN, Naples 80131, Italy; 5 Department of Respiratory Medicine, AO Monaldi, University of Naples Federico II, Via Leonardo Bianchi, Monaldi Hospital, Naples 80131, Italy; E-Mail: matsouni@libero.it; 6 Dipartimento di Scienze e Tecnologie Ambientali Biologiche Farmaceutiche, Seconda Università degli Studi di Napoli, Via Vivaldi 43, Caserta 81100, Italy

**Keywords:** COPD, adiponectin, metabolic disorders, adipose tissue

## Abstract

Chronic Obstructive Pulmonary Disease (COPD) is a chronic inflammatory lung disease which may be complicated by development of co-morbidities including metabolic disorders. Metabolic disorders commonly associated with this disease contribute to lung function impairment and mortality. Systemic inflammation appears to be a major factor linking COPD to metabolic alterations. Adipose tissue seems to interfere with systemic inflammation in COPD patients by producing a large number of proteins, known as “adipokines”, involved in various processes such as metabolism, immunity and inflammation. There is evidence that adiponectin is an important modulator of inflammatory processes implicated in airway pathophysiology. Increased serum levels of adiponectin and expression of its receptors on lung tissues of COPD patients have recently highlighted the importance of the adiponectin pathway in this disease. Further, *in vitro* studies have demonstrated an anti-inflammatory activity for this adipokine at the level of lung epithelium. This review focuses on mechanisms by which adiponectin is implicated in linking COPD with metabolic disorders.

## 1. Introduction

There is a growing awareness that chronic obstructive pulmonary disease (COPD) is a lung disease not only restricted to pulmonary inflammation and airway remodeling [1]. Systemic effects and extrapulmonary comorbidities are common and significantly impact health outcomes in patients, including mortality. Cardiovascular diseases, musculoskeletal disorders, diabetes mellitus II and metabolic syndrome are among the most prevalent comorbidities; however the underlying molecular basis linking COPD and comorbidities are still not fully understood [2,3,4] although, in the last years, the relationship between comorbidities and systemic inflammation in COPD has been extensively analyzed. Whether systemic inflammation in COPD has been well recognized, the genesis of the systemic involvement needs to be further explored. It remains unclear whether systemic inflammation is the result of systemic diffusion of local inflammation in the lungs, or is a consequence of comorbid conditions which impact on lungs. Understanding how lung inflammation in COPD produces systemic consequences and interferes with co-morbidity development should be a priority.

Some evidence indicates that airway inflammation is mediated through a variety of cells, including macrophages, neutrophils, and T and B lymphocytes by the secretion of a number of pro-inflammatory cytokines; moreover cell trafficking at epithelium level is mainly coordinated by adhesion molecules expression [5,6,7,8,9]. One of the major cell types involved in inflammatory events associated with COPD is alveolar macrophages, which are able to produce several chemotactic factors [10,11]. The high production of chemo-attractants results in increased lung neutrophil and lymphocyte infiltration which in turn produce pro-inflammatory cytokines that contribute to the development and progression of the lung disease. The chronicity of COPD systemic inflammation is sustained by an increased production of several pro-inflammatory cytokines at both serum and airway levels. Indeed, C-reactive protein (CRP), fibrinogen, IL1β, TNFα, MCP1, IL8, IL6 have been associated with disease progression and exacerbation [12,13,14], whilst an inverse correlation between anti-inflammatory cytokine IL-10 and COPD has been demonstrated.

The development of chronic systemic inflammation in COPD has not yet been fully understood. One possible mechanism may be due to the spill-over of pro-inflammatory factors and cells (neutrophils, lymphocytes) from lung to peripheral blood [15]. The contribution of lung in induction of this process is probably due to the increased permeability of pulmonary vessels in COPD, which contributes to the emission of pro-inflammatory. Another hypothesis supports the proposition that an enhanced expression of granulocyte-macrophage colony-stimulating factor and IL-6 in COPD plays an important role, as these mediators stimulate neutrophils release from the bone marrow causing an increased neutrophil amount in the peripheral blood [16,17]. However, it is notable that systemic inflammation is heterogeneously distributed over COPD patients [11] and that comorbidities may be related to age rather than disease severity [18]. Therefore, the precise mechanism is still not fully understood.

Airway epithelium represents a central site for the mechanisms involved in the complex interaction between environmental triggers, airway inflammation and metabolic pathways [12,13,14,19,20]. Regarding peripheral organs, muscle, liver and adipose tissues are a source of a wide range of inflammatory mediators that have systemic effects when released in the systemic compartment.

The systemic inflammatory state has also been attributed to metabolic impairments in COPD patients [2,10,11]. Recent clinical investigations have highlighted the role of metabolic syndrome biomarkers to predict lung function impairment [16]. Naaved *et al*. found that dyslipidemia, elevated heart rate, increased insulin resistance and leptin levels were independent risk factors of greater susceptibility to lung function impairment within six months of World Trade Center irritant exposure, whereas elevated amylin was protective [21]. The authors suggest that metabolic syndrome biomarkers expressed soon after exposure to World Trade Center dust predicted FEV1 decline, and the systemic inflammation produced by metabolic syndrome impacted on progression to abnormal lung function in a longitudinally followed cohort [21]. Through these observations, a growing interest has been focused on the interaction between metabolic disorders and COPD; however, it remains still to be elucidated which biological pathway is mainly involved.

The potential interaction between abnormal adipose tissue and lung function impairment may be ascribed to systemic inflammation, thus providing new insight into the pathogenesis and reversibility of systemic involvement of COPD [22]. Accordingly, a link between adipose tissues and circulating concentrations of TNFα, IL6, leptin and adiponectin that play an important part in metabolic changes associated with COPD and in lung function decrease has been demonstrated [23].

In particular, the importance of adiponectin expression in lung function and COPD severity has been recently highlighted. This review will focus on the mechanisms by which the adiponectin system is a potentially attractive marker for systemic involvement in COPD.

## 2. Adiponectin and Adipose Tissue

A key role for systemic inflammation in development of both metabolic disorders and lung function impairment has been recently reported and, therefore, scientific interest has been focused on adipocyte-derived cytokines including adiponectin. Adipose tissue is a highly dynamic endocrine organ that synthesizes and secretes a variety of proteins known as “adipokines” involved in several metabolic processes such as immunity, insulin resistance, lipid and glucose metabolisms and inflammation [21,22,23,24,25].

Adiponectin is known to exert anti-inflammatory effects. It is a proteic hormone synthetized and secreted by adipose tissue as a 30 kDa monomer that, due to post-translational modifications, forms characteristic homomultimers. In fact, adiponectin assembles into several oligomeric multimers including trimers, known as low molecular weight (LMW); hexamers, known as medium molecular weight (MMW) and higher-molecular weight (HMW) multimeric complexes [26]. Growing evidence associates the oligomerization process with multiple biological activities of adiponectin since HMW are considered the most biologically active isoforms [26,27]. Adiponectin structurally belongs to the complement 1q family and is found at high concentrations (>0.01% of the total protein) in serum of healthy individuals [28]. In humans, the gene encoding adiponectin (ACDC) is located on chromosome 3q27; single-nucleotide polymorphisms (SNPs) and haplotypes in ACDC gene have been associated with obesity as well as with metabolic syndrome and cardiovascular diseases [29,30,31,32]. Two receptors (AdipoR1 and AdipoR2), ubiquitous expressed in several organs, including lung, tissues and cell lines, mediate adiponectin effects [26,33,34]. Downstream of these two receptors, the biological effects of adiponectin are mediated by different signal pathways involving the following molecules: AMPK, ERK, AKT and P38 [33,35]. In particular, it is reported that AdipoR1 is mainly implicated in the metabolic functions of adiponectin, whereas AdipoR2 is more involved in anti-inflammatory and anti stress-oxidative activities [33,36,37].

In obesity, systemic concentrations of total adiponectin and its different isoforms are disproportionately reduced [38]. Moreover, the proportion of HMW isoforms to total adiponectin is lower among obese individuals than healthy controls [38]. One explanation for this surprising finding is that adipose tissue in the obese experiences localized hypoxia that inhibits the expression of adiponectin [38,39]. The hypoxia-induced necrosis of adipocytes attracts activated macrophages that collect to form functional syncytia surrounding the necrotizing adipocytes. These syncytia produce TNFα and IL6 which may inhibit the local production of adiponectin by the adipose tissue [24,40]. Interestingly, there are marked sexual differences of the distribution of adiponectin and compared to men, women have higher circulating levels of HMW isoforms, despite greater levels of overall adiposity. Moreover, postmenopausal women have higher proportions of HMW than premenopausal women, with a negative association of circulating concentrations of total and HMW adiponectin with sex steroids [41,42].

## 3. Adiponectin, Metabolic Disorders and COPD

### 3.1. Adiponectin Role in Inflammation

Adiponectin is important in energy homeostasis, regulating both glucose and lipid metabolism. In humans, down regulation of adiponectin and its receptors is associated with obesity, metabolic syndrome, insulin resistance, hyperinsulinemia and type 2 diabetes, as well as with cardiovascular diseases [26,31]. Moreover, adiponectin seems implicated in the development and progression of several local and systemic inflammatory processes.

In fact, it has been recently outlined that adiponectin could play an important role in anti-inflammatory responses in several tissues and cell cultures such as pancreatic beta cells and endothelial cells [43,44,45]. A protective anti-inflammatory role of HMW oligomers has been demonstrated both *in vivo* and *in vitro* studies [26,36,46,47]. Research findings indicate that adiponectin exerts its anti-inflammatory properties by inhibiting several pro-inflammatory mediators (TNFα, IL6, endothelial adhesion molecules ICAM-1 and nuclear factor-κB) [48,49] and promoting anti-inflammtory mediators (IL10 and IL1 receptor antagonist) [50]. *In vitro* studies show the additional down-regulation of the inflammatory infectious state of adiponectin while binding bacterial lipoplysaccharides [51]. In addition, HMW oligomers seem to improve insulin sensitivity, suppress apoptosis in endothelial cells and are inversely correlated to cardiovascular events and to the severity of coronary artery disease [46]. The role of adiponectin in energy metabolism has been widely studied, while little is known about its role in inflammatory processes of lung [24,52]. In fact, human and murine studies are still inconclusive and both pro-inflammatory and anti-inflammatory lung adiponectin properties have been hypothesized.

### 3.2. Evidence for a Role of Adiponectin in COPD: Human Studies

Human studies have demonstrated a significant increase of serum adiponectin levels in COPD and a direct correlation to the severity of the disease [24,52,53]. Indeed, relationship between adiponectin concentrations and accelerated decline in lung functions has been reported [54,55,56]. Moreover, serum adiponectin levels are not significantly influenced by smoking status in COPD [54]. More recently, Carolan *et al*. reported adiponectin contribution also in development of emphysema [57].

Serum adiponectin levels represent a significant diagnostic and prognostic marker of COPD but the real biological effects of adiponectin and of its oligomers on human lung and even less in lung diseases are not fully clear [54,55,56]. Recently, it has demonstrated that the oligomerization pattern of adiponectin is altered in COPD; in particular the higher levels of adiponectin are associated with a specific increase of HMW, the most biologically active isoforms [58]. Daniele *et al*. found no detectable TNFα values in normal subjects and in COPD patients suggesting that the high levels of adiponectin and HMW could be involved in reducing the increase of circulating levels of this pro-inflammatory cytokine [58].

Emerging evidence suggests adiponectin as an immunomodulator and tumor adipokine since it is implicated in the pathogenesis and prognosis of different cancers, including lung cancer [58,59]. In fact, it was demonstrated that both AdipoRs are expressed in COPD and tumor lung [33,59]; moreover a recent study demonstrated a higher expression of AdipoR1 than AdipoR2 in lung tissues from COPD and non small cell lung cancer (NSCLC) suggesting a specific signaling pathway of adiponectin in this disease [60]. In addition, the down-regulation of AdipoR2 could be responsible for the worsening of the inflammatory state in COPD and related to the development of lung NSCLC cancer [60]. Recent studies furthermore indicate an association between genetic variants in adiponectin gene and COPD as well as in NSCLC [61,62]. Recently, Ghafar *et al*. suggested that adiponectin could suppress NSCLC cancer cell development by AdipoR2 while AdipoR1 may have favorable prognostic significance [63]. The molecular mechanism underlying adiponectin effects on normal cells remains not yet completely clarified. A recent *in vitro* study evaluated the effects of adiponectin on A549 cells, a model of alveolar epithelium showing that, in a time and dose-dependent manner, it decreases cell viability and increases apoptosis through ERK1/2 and AKT [36].

### 3.3. Anti-Inflammatory Role in Lung and COPD (*in Vitro* and *Vivo* Studies)

The secretion of several inflammatory molecules, such as IL1β and TNFα, from different cell types is increased by reactive oxygen species. In particular, TNFα and IL1β induce both macrophages to secrete matrix metalloproteinase-9, and bronchial epithelial cells to produce extracellular matrix can increase gene expression of glycoproteins. Recent data have revealed the anti-inflammatory role in the lungs of genetically-induced adiponectin deficient mice (Adiponectin^−/−^), showing higher expression of TNFα in alveolar macrophages and abnormal alveolarization, resembling an emphysema-like phenotype, reversible with adiponectin supplementation [49,64]. The anti-inflammatory properties of adiponectin are further supported by extrapulmonary inflammation, vascular endothelial dysfunction and some other comorbidities (osteoporosis and cachexia) shown in the same mouse model [49,64]. Furthermore, Summer *et al*. hypothesized a protective role of adiponectin on the lungs through inhibition of alveolar macrophage function and vascular homeostasis regulation [49,64]. Moreover, Nigro *et al*. [36] observed that, in cells exposed to TNFα and/or IL1β, two potent lung inflammatory cytokines, adiponectin, in dose- and time-dependent manner, reduce cytotoxic effects of TNFα and IL1β improving cell viability and decreasing apoptosis. In the same paper, it was demonstrated that adiponectin inhibits NF-κB nuclear trans-activation and induces the expression of the anti-inflammatory IL10 cytokine via ERK1/2 and AKT through the mediation of AdipoR1 [36].

### 3.4. Pro-Inflammatory Role in Lung and COPD (*in Vitro* and *Vivo* Studies)

On the other hand, it was reported that adiponectin may induce inflammatory activation in macrophages and in A549 cells [65]; moreover, in adiponectin-deficient mice, it was demonstrated that adiponectin could play an important pro-inflammatory role in tobacco smoke-induced COPD [66].

However, why adiponectin has been found to exhibit pro-inflammatory effects under certain conditions needs to be investigated.

In conclusion, although the biological meaning of adiponectin modulation remains to be clarified, a protective role of adiponectin and its oligomeric isoforms in lung inflammation, through AdipoR1 receptor has been widely demonstrated. It is not completely clear how modulation of adiponectin, may be helpful in COPD prevention and treatment. However, most evidence demonstrate that adiponectin represents a useful diagnostic and prognostic biomarker in COPD. Therefore, further studies are needed to fully elucidate the complexity of adiponectin system effects in the lungs.

## 4. Conclusions

In this review, the role of adiponectin system in lung disorders has been analyzed. Investigations on adiponectin systemic production and epithelial cell receptors at lung level have provided new insight in COPD pathophysiology. Serum levels of adiponectin represent a significant diagnostic and prognostic marker for COPD disease. The molecular mechanisms through which adiponectin mediates its effects in the lungs are not clearly defined; however, AdipoRs expression on lung epithelial cells of COPD patients strengthens the hypothesis of its role in pathophysiological conditions of the lungs.

Altogether, the reported evidence indicate that adiponectin and its oligomerization state are involved in lung diseases and that its increased levels are mainly due to HMW oligomers. The observations reported suggest that total levels of adiponectin, HMW oligomers and AdipoRs could respresent useful complementary criteria to improve therapeutic and prognostic strategies for diseases that involve lung impairment. Adiponectin seems to exert a direct protective role on lung epithelial A549 cells exhibiting anti proliferative and anti-inflammatory effects. Furthermore, *in vitro* studies demonstrated that adiponectin protects A549 lung cells against cytotoxicity induced by TNFα- or IL-1β.

Better understanding of mechanisms underlying systemic inflammation and extrapulmonary comorbidities in COPD will help identification of new targets for both diagnostic and therapeutic approaches. Adiponectin appears to be an attractive biomarker in COPD and represents a promising disease indicator with potential implications in COPD therapeutical management.
